# How type 2 diabetes mellitus shapes carotid artery changes in hemorrhagic and ischemic stroke patients: a comparative analysis of carotid intima-media thickness and pulsatility index

**DOI:** 10.11604/pamj.2026.53.37.50343

**Published:** 2026-01-27

**Authors:** Chairil Amin Batubara, Diko Hamonangan Saragih, Justika Usmadhani Aulya, Cut Aria Arina

**Affiliations:** 1 Department of Neurology, Faculty of Medicine, Universitas Sumatera Utara, Medan, Indonesia,; 2 Faculty of Medicine, Universitas Sumatera Utara, Medan, Indonesia

**Keywords:** Intima-media thickness, pulsatility index, stroke, type 2 diabetes mellitus

## Abstract

Stroke is the second leading cause of death globally and a major cause of disability. Type 2 diabetes mellitus worsens atherosclerosis and raises stroke risk. Intima-media thickness (IMT) and pulsatility index (PI) measurements of the common carotid artery are valuable markers to detect atherosclerosis and hemodynamic changes in stroke patients, especially those with T2DM. We aim to compare IMT and PI in stroke patients with and without T2DM at Haji Adam Malik General Hospital, Medan. This study employed an analytical observational design with a cross-sectional approach. The subjects included ischemic and hemorrhagic stroke patients who met the inclusion and exclusion criteria. IMT and PI measurements were performed using Doppler ultrasonography. Data were analyzed using comparative statistical tests to assess differences between stroke patients with and without T2DM. The mean intima-media thickness (IMT) of the common carotid artery was significantly higher in stroke patients with T2DM compared to those without T2DM (1.55±0.23 mm vs. 0.88±0.28 mm; p<0.001). The pulsatility index (PI) was also significantly higher in stroke patients with T2DM (2.00±0.32 vs. 1.39±0.12; p<0.001), although some variation was observed depending on the type of stroke (ischemic or hemorrhagic). These differences indicate increased arterial stiffness and cerebrovascular resistance in stroke patients with T2DM. There are significant differences in intima-media thickness (IMT) and pulsatility index (PI) of the common carotid artery between stroke patients with and without Type 2 diabetes mellitus. IMT and PI measurements may serve as non-invasive indicators to assess the severity of atherosclerosis and hemodynamic changes in stroke patients, particularly those with comorbid T2DM.

## Introduction

Stroke is the second leading cause of death globally and contributes significantly to morbidity and mortality. It is also the third leading cause of disability worldwide [1,2]. Carotid atherosclerosis is a key factor in arterial embolism, accounting for 15-20% of ischemic strokes. Other studies suggest that up to 28% of ischemic strokes are caused by atherothrombotic events, with carotid stenosis being the primary culprit. Up to 43% of patients with atherothrombotic strokes experience a preceding transient ischemic attack (TIA). The risk of recurrence is also high in patients with atherothrombotic stroke, making the evaluation and management of arterial stenosis crucial. Unstable or ruptured plaques at the bifurcation of the common carotid artery or the proximal internal carotid artery trigger thrombus formation, which can dislodge and cause distal arterial occlusion, leading to TIA symptoms. Studies have shown that the degree of carotid artery stenosis is a crucial predictor of stroke risk [1,3]. Doppler ultrasonography is used to assess intima-media thickness (IMT), stenosis, plaques, lumen abnormalities, and vascular wall changes. It is a safe, affordable, reliable, repeatable, and convenient tool for detecting carotid atherosclerosis. IMT, or carotid intima-media thickness (CIMT), is measured as the thickness between the intimal-luminal and medial-adventitial layers. CIMT has been identified as a strong predictor of future vascular events, such as myocardial infarction and stroke, and is considered a predictor of future strokes [1,4].

Pulsatility index (PI) is a reliable indicator of distal cerebrovascular resistance and correlates with cerebral perfusion pressure. PI is closely linked to arterial stiffness and vascular resistance, both of which play significant roles in reducing pulsatile transmission to the microcirculation of end organs [5,6]. A high PI in the common carotid artery is associated with aging, hypertension, high-grade stenosis, and increased severity on the National Institutes of Health Stroke Scale (NIHSS). Age and hypertension are also linked to higher PI values, indicating vascular resistance in stroke patients. The PI of the common carotid artery provides a more accurate index for assessment, as it is less dependent on the examiner and is independent of the insonation angle [7]. Diabetes mellitus (DM) is a major risk factor for cerebrovascular disease. Patients with DM have a higher mortality rate from stroke and experience more severe and progressive atherosclerosis. A study by Bill *et al*. found that stroke patients with DM had higher cerebrovascular risk factors and more significant findings on duplex sonography, including increased intima-media thickness (IMT) and a higher PI in the common carotid artery. The study also showed that these two parameters are independently associated with DM [7,8]. Therefore, this study aims to assess the differences in intima-media thickness and pulsatility index of the common carotid artery in stroke patients with and without type 2 DM at H. Adam Malik General Hospital, Medan.

## Methods

This analytical observational study with a cross-sectional design was conducted at the Stroke Corner and Integrated Inpatient Unit of Haji Adam Malik General Hospital (RSUP HAM) Medan from August 2023 to March 2025. The subjects were all acute stroke (ischemic and hemorrhagic) patients with and without type 2 DM who met the inclusion and exclusion criteria. The sample size was determined using the formula for unpaired numerical analytical studies, yielding a total of 36 subjects. Inclusion criteria included acute-phase ischemic stroke or hemorrhagic stroke confirmed by head CT scan, age above 18 years, and informed consent to participate in the study. Exclusion criteria included posterior circulation stroke, recurrent stroke, traumatic intracerebral hemorrhage, clinical deterioration with an Early Warning Score (EWS) =7, chronic kidney disease, or a history of carotid endarterectomy.

Pulsatility index (PI) was defined as the difference between peak systolic velocity (PSV) and end-diastolic velocity (EDV), divided by the mean flow velocity (MFV) over the cardiac cycle. Measurements were taken 2 cm distal to the carotid bifurcation using Doppler ultrasonography (Vinno M80). Carotid intima-media thickness (CIMT) was defined as the thickness between the intimal-luminal and medial-adventitial layers, assessed using Doppler ultrasonography (Vinno M80) in the common carotid artery on the side of the stroke lesion, 2 cm from the bifurcation. Subjects who met the inclusion and exclusion criteria underwent Doppler ultrasonography to assess PI and CIMT, followed by data analysis. Univariate analysis was performed to describe the overall subject characteristics. Bivariate analysis using the independent t-test was conducted to evaluate differences in PI and CIMT between stroke patients (ischemic and hemorrhagic) with and without type 2 DM. Data analysis was performed using Windows Statistical Product and Service Solutions (SPSS) version 22.0. This study was approved by the Ethics Committee of the Faculty of Medicine, University of North Sumatra.

## Results

A total of 36 subjects were included, consisting of 18 ischemic stroke patients and 18 hemorrhagic stroke patients. The majority of subjects were male (58.3%), with a mean age of 55.29 ± 10.26 years. The mean age of ischemic stroke patients (56.33 ± 11.11 years) was higher than that of hemorrhagic stroke patients (54.44 ± 9.56 years). When categorized by stroke type, ischemic stroke was more common in males (61.1%) than females (38.9%), whereas hemorrhagic stroke had a nearly equal gender distribution (55.6% males and 44.4% females). The demographic characteristics of the study subjects are presented in Table 1. Table 2 presents the effect of diabetes mellitus (DM) on the pulsatility index (PI) in patients with hemorrhagic and ischemic stroke. In hemorrhagic stroke patients, the mean PI in the DM group was higher (2.00 ± 0.32) compared to the non-DM group (1.39 ± 0.12). This difference was statistically significant (p < 0.001), with a mean difference of 0.60 (95% CI: 0.3611-0.8500), indicating that the presence of DM significantly contributes to an increased PI in patients with hemorrhagic stroke.

**Table 1:** characteristics of subjects

Characteristics	n (%)	
	Ischemic stroke	Hemorrhagic stroke
**Gender (n, %)**		
Male	11 (61.1%)	10 (55.6%)
Female	7 (38.9%)	8 (44.4%)
**Ethnicity (n, %)**		
Bataknese	11 (61.1%)	11 (61.1%)
Javanese	2 (11.1%)	4 (22.2%)
Aceh	1 (5.6%)	2 (11.1%)
Malay	3 (16.6%)	1 (5.6%)
Padang	1 (5.6%)	0 (0%)
Age* (mean ± SD)	56.33 ± 11.114	54.44 ± 9.568
**Education (n, %)**		
Elementary School	3 (16.6%)	1 (5.6%)
Junior High School	2 (11.1%)	4 (22.2%)
Senior High School	8 (44.4%)	9 (50%)
Bachelor	5 (27.9%)	4 (22.2%)
**Occupation (n, %)**		
Teacher	1 (5.6%)	1 (5.6%)
Entrepreneur	8 (44.4%)	8 (44.4%)
Housewife	2 (11.1%)	5 (27.8%)
Private Employee	3 (16.6%)	3 (16.6%)
Retiree	2 (11.1%)	1 (5.6%)
No occupation	2 (11.1%)	0 (0%)
CIMT level* (mean ± SD)	1.38 ± 0.33	1.22 ± 0.43
PI level* (mean ± SD)	1.67 ± 0.46	1.69 ± 0.39

* Normally distributed data

**Table 2:** difference in pulsatility index in stroke patients with and without T2DM

Type of stroke	Pulsatility Index (mean±SD)	P-value
**Ischemic stroke**		
With DM	2.05 ± 0.26	<0.001
Without DM	1.28 ± 0.20	
**Hemorrhagic stroke**		
With DM	2.00 ± 0.32	<0.001
Without DM	1.39 ± 0.12	

Similarly, in ischemic stroke patients, the mean PI was also higher in the DM group (2.05 ± 0.26) compared to the non-DM group (1.28 ± 0.20). This difference was statistically significant (p < 0.001), with a mean difference of 0.76 (95% CI: 0.5285-1.0004), indicating a strong effect size and confirming that DM has a significant impact on increasing PI in ischemic stroke patients. Table 3 illustrates the effect of DM on carotid intima-media thickness (CIMT) in patients with hemorrhagic and ischemic stroke. In hemorrhagic stroke patients, the mean CIMT was higher in the DM group (1.55 ± 0.23) compared to the non-DM group (0.88 ± 0.28). This difference was statistically significant (p < 0.001), with a mean difference of 0.66 (95% CI: 0.4081 - 0.9252), indicating that DM strongly impacts CIMT elevation in hemorrhagic stroke patients. Likewise, in ischemic stroke patients, the mean CIMT in the DM group was higher (1.63 ± 0.23) compared to the non-DM group (1.12 ± 0.17). This difference was also statistically significant (p < 0.001), with a mean difference of 0.51 (95% CI: 0.3110 - 0.7223), demonstrating that DM significantly contributes to increased CIMT in ischemic stroke patients.

**Table 3:** difference in CIMT in stroke patients with and without T2DM

Type of stroke	CIMT (mean±SD)	Nilai P
**Ischemic stroke**		
With DM	1,63 ± 0,23	<0.001
Without DM	1,12 ± 0,17	
**Hemorrhagic stroke**		
With DM	1,55 ± 0,23	<0.001
Tanpa DM	0,88 ± 0,28	

## Discussion

The demographic characteristics of this study provide insight into the potential influences of sex, age, and diabetes mellitus on carotid intima-media thickness (IMT) and pulsatility index (PI) among stroke patients. Of 36 subjects, males predominated (58.3%), consistent with previous findings that male gender increases stroke risk due to higher rates of hypertension, smoking, and alcohol use [9]. Estrogen provides vascular protection in premenopausal women; however, the loss of estrogen after menopause increases susceptibility to stroke [7]. Males generally have higher IMT due to greater exposure to atherosclerotic factors, whereas diabetic females tend to show greater increases in IMT and PI, indicating that hyperglycemia accentuates arterial stiffness [9,10]. The mean participant age was 55.39 ± 10.27 years, with ischemic stroke patients being older than hemorrhagic ones. Advancing age contributes to vascular stiffness and reduced elasticity, leading to elevated IMT and PI, thereby impairing cerebrovascular autoregulation [7]. In diabetes, chronic hyperglycemia accelerates these processes, promoting atherosclerosis and worsening perfusion [10]. Age-related vascular changes, compounded by hypertension and hormonal decline, heighten both ischemic and hemorrhagic stroke risk [11].

Mean PI was 1.68 ± 0.41, and mean CIMT was 1.30 ± 0.38 mm, higher in ischemic than hemorrhagic stroke. This aligns with Nasser *et al*. who found thicker carotid IMT in ischemic stroke, reflecting more advanced atherosclerosis [4]. Although limited research compares PI between stroke types, our finding of slightly higher PI in hemorrhagic stroke may reflect elevated intracranial pressure, which correlates with increased PI [12]. Diabetes mellitus (DM) substantially impacts cerebrovascular structure and hemodynamics. Studies consistently show that diabetic stroke patients exhibit higher IMT and PI due to increased vascular resistance and atherosclerotic changes [7,13]. Higher PI in diabetic stroke reflects impaired perfusion and worse outcomes [14,15]. In our cohort, both ischemic and hemorrhagic stroke patients with diabetes had significantly higher PI than non-diabetic counterparts (p < 0.001), consistent with Bill *et al*. and Das *et al*. [7,10,14,15]. These results emphasize diabetes as a strong determinant of cerebrovascular resistance and stroke severity.

PI is an important indicator of vascular stiffness and stroke prognosis, particularly in diabetes, which accelerates cerebrovascular aging and microangiopathy [7,14]. Elevated PI, especially in the basilar artery, signifies small-vessel disease and poor collateral circulation [15]. High PI also correlates with NIHSS scores and unfavorable outcomes in both ischemic and hemorrhagic stroke [14,16]. Moreover, in diabetic patients, PI reflects systemic vascular pathology and can help distinguish focal from generalized vascular resistance [15]. Our study also identified significantly higher PI in hemorrhagic stroke patients with diabetes (p < 0.001). Prolonged hyperglycemia causes endothelial dysfunction, arterial stiffness, and increased pulsatility [17,18]. This is further aggravated by elevated intracranial pressure and microangiopathy, which together worsen perfusion and vascular resistance [7,18]. Hence, PI may serve as a non-invasive marker for cerebrovascular burden in diabetic stroke.

Intima-media thickening is a recognized marker of atherosclerosis and a predictor of thrombotic stroke, particularly in diabetes [7,13]. Endothelial dysfunction and inflammation in type 2 DM accelerate IMT progression and increase ischemic stroke risk [13]. Our results concur with Bill *et al*. who reported significantly higher CIMT in ischemic stroke patients with diabetes [7,11], and with Guo *et al*. who found elevated IMT correlated with poor outcomes on the modified Rankin Scale [19]. Elevated PI also indicates worse hemodynamic disturbance and risk of neurological deterioration [18]. Carotid IMT > 0.8 mm strongly predicts ischemic stroke, with incidence approaching 100% at IMT ≥ 1.1 mm [13]. Plaque formation is markedly more common in diabetic stroke patients (83.3%) than in diabetics without stroke (16.7%) [13]. Although increased IMT is mainly linked to ischemic stroke, studies also show elevated IMT in hemorrhagic cases, possibly due to hypertensive vascular remodeling [4,20]. Ohira *et al*. demonstrated that individuals with high IMT have increased hemorrhagic stroke risk (RR 2.55, 95% CI 1.09-5.94) [20].

Our finding of higher CIMT in hemorrhagic stroke patients with diabetes suggests that hyperglycemia-driven endothelial injury and oxidative stress accelerate vascular wall thickening [21,22]. These mechanisms may contribute to vessel fragility and predispose to hemorrhage. This study´s limitations include a small sample size, absence of analysis on stroke duration or comorbid risk factors, and lack of follow-up data to evaluate IMT and PI progression post-treatment.

## Conclusion

There are significant differences in intima-media thickness (IMT) and pulsatility index (PI) of the common carotid artery between stroke patients with and without Type 2 diabetes mellitus. IMT and PI measurements may serve as non-invasive indicators to assess the severity of atherosclerosis and hemodynamic changes in stroke patients, particularly those with comorbid T2DM.

### What is known about this topic

- There are significant differences in intima-media thickness (IMT) and pulsatility index (PI) of the common carotid artery between stroke patients with and without T2DM;
- IMT and PI measurements may serve as non-invasive indicators to assess the severity of atherosclerosis and hemodynamic changes in stroke patients, particularly those with comorbid T2DM.

### What this study adds

- The mean intima-media thickness (IMT) and the pulsatility index (PI) of the common carotid artery were significantly higher in stroke patients with T2DM compared to those without T2DM;
- These differences indicate increased arterial stiffness and cerebrovascular resistance in stroke patients with T2DM.
